# Shared Pathways Among Autism Candidate Genes Determined by Co-expression Network Analysis of the Developing Human Brain Transcriptome

**DOI:** 10.1007/s12031-015-0641-3

**Published:** 2015-09-23

**Authors:** Ahmed Mahfouz, Mark N. Ziats, Owen M. Rennert, Boudewijn P.F. Lelieveldt, Marcel J.T. Reinders

**Affiliations:** Delft Bioinformatics Lab, Delft University of Technology, Delft, The Netherlands; Department of Radiology, Leiden University Medical Center, Leiden, The Netherlands; Department of Intelligent Systems, Delft University of Technology, Delft, The Netherlands; National Institute of Child Health and Human Development, National Institutes of Health, Bethesda, MD USA; University of Cambridge, Cambridge, UK; Baylor College of Medicine, Houston, TX USA

**Keywords:** Autism spectrum disorder, Gene co-expression network, Synaptogenesis, Mitochondrion, Apoptosis

## Abstract

Autism spectrum disorder (ASD) is a neurodevelopmental syndrome known to have a significant but complex genetic etiology. Hundreds of diverse genes have been implicated in ASD; yet understanding how many genes, each with disparate function, can all be linked to a single clinical phenotype remains unclear. We hypothesized that understanding functional relationships between autism candidate genes during normal human brain development may provide convergent mechanistic insight into the genetic heterogeneity of ASD. We analyzed the co-expression relationships of 455 genes previously implicated in autism using the *BrainSpan* human transcriptome database, across 16 anatomical brain regions spanning prenatal life through adulthood. We discovered modules of ASD candidate genes with biologically relevant temporal co-expression dynamics, which were enriched for functional ontologies related to synaptogenesis, apoptosis, and GABA-ergic neurons. Furthermore, we also constructed co-expression networks from the entire transcriptome and found that ASD candidate genes were enriched in modules related to mitochondrial function, protein translation, and ubiquitination. Hub genes central to these ASD-enriched modules were further identified, and their functions supported these ontological findings. Overall, our multi-dimensional co-expression analysis of ASD candidate genes in the normal developing human brain suggests the heterogeneous set of ASD candidates share transcriptional networks related to synapse formation and elimination, protein turnover, and mitochondrial function.

**Electronic supplementary material:**

The online version of this article (doi:10.1007/s12031-015-0641-3) contains supplementary material, which is available to authorized users.

## Introduction

Autism spectrum disorder (ASD) is a neurodevelopmental syndrome characterized clinically by impairments in verbal and non-verbal communication, deficits in social interaction, and repetitive and/or restrictive patterns of behavior (American Psychiatric Association 2000). Despite an estimated prevalence of 1 in 88 newborns (Centers for Disease Control and Prevention 2012), and an exponential increase in recent efforts to elucidate autism neurobiology, a clear understanding of the molecular mechanisms underlying the development of ASD remains elusive. However, recent studies have firmly established a substantial role for genetic etiologies in the development of ASD. Evidence for a strong heritable risk of ASD was initially described in twin and sibling epidemiological studies of autism (Smalley et al. 1988; Ritvo et al. 1989; Steffenburg et al. 1989; Bailey et al. 1995; Hallmayer et al. 2011), and has since been firmly established through multiple genetic approaches (Geschwind 2011; Berg and Geschwind 2012, Krumm et al. 2014). For instance, genome-wide association studies (GWAS) (Wang et al. 2009; Weiss et al. 2009; Anney et al. 2010), copy number variation (CNV) analysis (Sebat et al. 2007; Szatmari et al. 2007; Marshall et al. 2008; Pinto et al. 2010; Levy et al. 2011; Sanders et al. 2011), and whole-exome sequencing projects (O’Roak et al. 2012; Sanders et al. 2012; Iossifov et al. 2012; Neale et al. 2012; De Rubeis et al. 2014; Iossifov et al. 2014) have implicated hundreds of genes in ASD. Yet, understanding how this diverse set of genes relates to the underlying molecular mechanisms and subsequent neuropathology of ASD is still unclear.

Mechanistic understanding of how ASD candidate genes relate to the neurobiology of autism is a difficult task, since genes encode multiple highly complex functions at different stages of development and across different regions of the brain. Moreover, the set of genes implicated in ASD is highly heterogeneous, and many of their functions are completely unknown. Furthermore, understanding how disruption in different genes with disparate functions still results in a common clinical phenotype makes developing common targeted biomarkers and treatments for ASD challenging. Therefore, in addition to attempts to identify genes that are causative for ASD, it is important to understand how ASD candidate genes may relate to each other during human neurodevelopment in order to identify potential shared molecular pathways.

One validated approach to integrate heterogeneous gene sets, in order to uncover shared molecular mechanisms, is through the analysis of gene co-expression patterns, which invokes the guilt-by-association heuristic that is pervasive in genomics research (Stuart et al. 2003; Wolfe et al. 2005). Several studies have demonstrated that genes with similar brain co-expression patterns are likely to function together in common cellular pathways (Oldham et al. 2008; Winden et al. 2009). These transcriptional co-expression relationships are particularly relevant to neurodevelopment, as the precise regulation of gene expression across brain regions at different ages instructs the exquisite specialization and connectivity within the brain. Since neurodevelopmental disorders such as autism are believed to result from functional aberrations within brain regions and/or disruption of inter-regional connectivity between regions (Geschwind and Levitt 2007), investigating the gene expression profiles of autism candidate genes across brain regions and throughout normal human neurodevelopment may provide insight into the complex functional genomics of this neurodevelopmental disorder.

A global survey of ASD gene co-expression patterns across normal human neurodevelopment could therefore facilitate our translation of ASD candidate genes to ASD candidate pathways, but this has not yet been undertaken. A recent study that assessed autism gene co-expression patterns in two adult human brains is an important step toward this goal (Ben-David and Shifman 2012b), but as autism is a neurodevelopmental disorder, it is imperative to understand the relationship of autism candidate genes in a developmental context. Conversely, other studies have explored the expression profiles of individual ASD candidates in human brain development (Kang et al. 2011) but lack an assessment of the relationships among these ASD candidates and how they relate to global transcriptional pathways important in brain development.

Transcriptome-based studies of the developing human brain have previously been limited in the sample size, number of brain structures analyzed, and developmental time points assessed, hampering the ability to evaluate the genetic contributors to neurodevelopmental disease comprehensively (Sun et al. 2005; Abrahams et al. 2007; Johnson et al. 2009; Ip et al. 2010; Somel et al. 2010). However, the recent availability of broad developmental surveys of gene expression, which cover many brain regions over multiple developmental stages, can greatly facilitate such analysis (Kang et al. 2011). The *BrainSpan* Transcriptional Atlas of the Developing Human Brain is a repository of RNA-seq expression profiling of 16 brain structures spanning early pre-natal development (8 weeks postconception) to adulthood (40 years of age). This publicly available atlas presents a unique opportunity to understand the spatial and temporal specificity of ASD candidate genes.

A few studies have recently assessed for co-expression relationships between subsets of autism-related genes and/or certain developmental windows using human brain gene expression relationships. For instance, Parikshak et al. analyzed the co-expression of autism and intellectual disability risk genes in neocortex and among cortical laminae from samples representing early development using weighted gene co-expression network analysis (WCGNA). They demonstrated that ASD risk genes were enriched in modules related to transcription and synaptic development, and furthermore that enriched in superficial cortical layers and glutamatergic projection neuron (Parikshak et al. 2013). Willsey et al. studied co-expression networks derived from nine genes harboring recurrent de novo loss-of-function mutations in autism patients and showed principally that the autism risk gene expression is most prominent in layer 5/6 cortical projection neurons during mid-fetal gestation (Willsey et al. 2013). Finally, using a different computational approach, Hormozdiari et al. integrated co-expression networks and protein-protein interaction networks of autism and intellectual disability risk genes identified in a recent cohort of 116 patients, and also showed that the autism genes enrich into networks related to transcription and synaptogenesis (Hormozdiari et al. 2015). Despite the importance of these results and their largely overlapping findings, no study has yet assessed very broad sets of autism risk genes across all brain regions and development time points to gain insight into potentially shared molecular pathways or affected brain regions among the incredibly heterogeneous autism genetic subtypes.

Here, we present an analysis of the spatial-temporal co-expression of ASD candidate genes across the normal developing human brain using the *BrainSpan* atlas. We developed a biologically driven computational approach to deduce functional relationships among this diverse set of genes. We first discovered modules of ASD candidates with biologically relevant temporal co-expression dynamics. These modules were related to the processes of synaptogenesis, apoptosis, and the neurotransmitter γ-aminobutyric acid (GABA). Then, we created a transcriptome-wide co-expression network from all genes expressed in the brain, to discover significant “molecular interaction modules” and demonstrated that ASD candidate genes are enriched only in modules related to the processes of synaptogenesis, mitochondrial function, protein translation, and ubiquitination. Lastly, we identified hub genes within the ASD-enriched molecular interaction modules, whose functions supported our ontological results, and which may serve as additional ASD candidate genes. Our analysis of this multi-dimensional expression data suggests pathways previously independently implicated in autism are related to each other through shared neurodevelopmental transcriptional networks.

## Results

### Spatio-temporal Gene Co-expression Analysis of ASD Candidate Genes

**Fig. 1:**
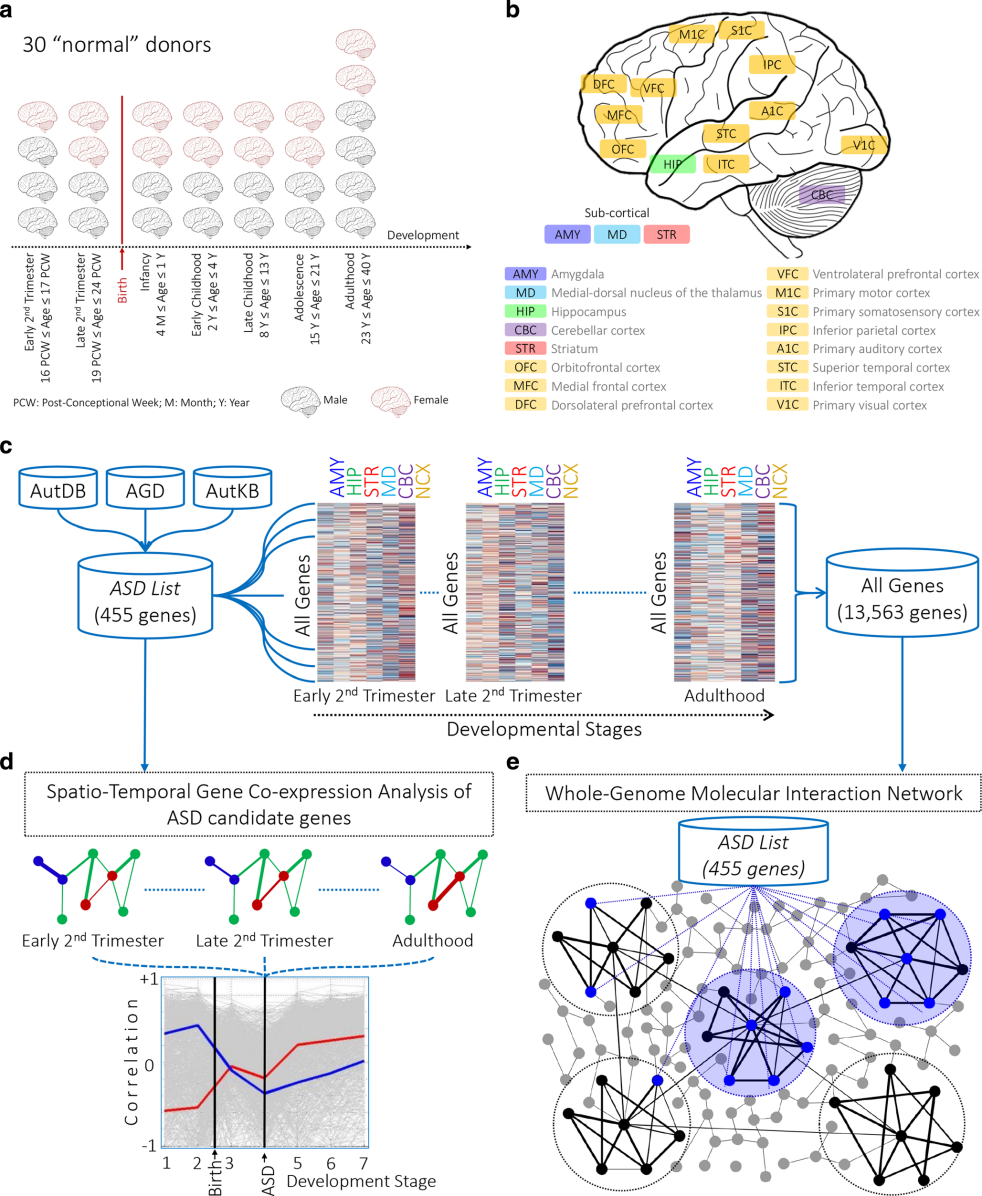
Analyzing ASD candidate genes in the *BrainSpan Atlas*. **a** Temporal description (i.e., age points) of the number and sex of the assessed brains. The data were grouped into seven developmental stages based on age. *Black-colored brains* indicate male donors, and *red-colored brains* indicate female donors. **b** A representation of the 16 structures sampled in the *BrainSpan Atlas*. **c** Each heatmap shows the expression of all genes across six representative brain regions (AMY, HIP, STR, MD, CBC, and NCX) in three representative developmental stages. The ASD list was created by combining lists of ASD candidate genes from three sources (AutDB, AGD, and AutKB-484). **d** A co-expression network of ASD candidate genes was generated for each developmental stage by correlating the expression vectors across brain regions. The correlation between each gene pair was tracked over the developmental stages. The *blue gene pair* represents two genes that are moderately correlated at early developmental stages but gain correlation through development. Stronger correlation is represented by a thicker edge between the two nodes. By contrast, the *red gene pair* represents two genes that lose correlation over development. *Bottom*, the correlation patterns of all gene pairs in the network (*gray*) across development. Correlation patterns of the blue and red pairs are shown in respective colors. Birth and the average age of ASD diagnosis are indicated. **e** The transcriptome-wide molecular interaction network was constructed based on the pairwise correlation between each pair of genes expressed in the *BrainSpan Atlas* (13,563 genes). Each node in the network represents a gene while the weighted edges represent correlations between genes based on their expression across all samples. Nodes were clustered into modules (*dashed circles*). Genes from the ASD list are highlighted within each module (*blue nodes*). *Blue circles* indicate modules that are significantly enriched in genes from the ASD list

In order to identify functional relationships between ASD candidate genes, we investigated patterns of gene co-expression change across developmental stages between each pair of genes from the *ASD list*. First, the correlation between each pair of ASD genes was calculated *separately* within each developmental stage based on the Spearman’s rank correlation between the two genes across all brain regions. For each gene pair, this resulted in a correlation value for each of the seven developmental stages, representing the brain-wide transcriptional similarity between the genes at each developmental stage (Fig. 1c, d). Gene pairs were retained only if they had an absolute correlation value *greater than 0.8* in at least one developmental stage. We have used the Spearman’s rank correlation as it focuses more on the similarity in the change of gene expression; as opposed to similarity in the absolute values of gene expression (see the Supplementary Information for more details).

Second, the surviving gene pairs were hierarchically clustered into distinct modules based on the similarity of their correlation profiles over time (using the Euclidean distance between the profiles and a complete linkage to merge clusters). Finally, the correlation pattern for each module was summarized by averaging all the gene pair correlation patterns included in the respective module. It is worth noting that the patterns within the modules represent changes in co-expression across development (which should not be confused with actual expression levels of genes).

#### ASD Gene Modules Display Distinct Temporal Dynamics Around Birth

**Fig. 2:**
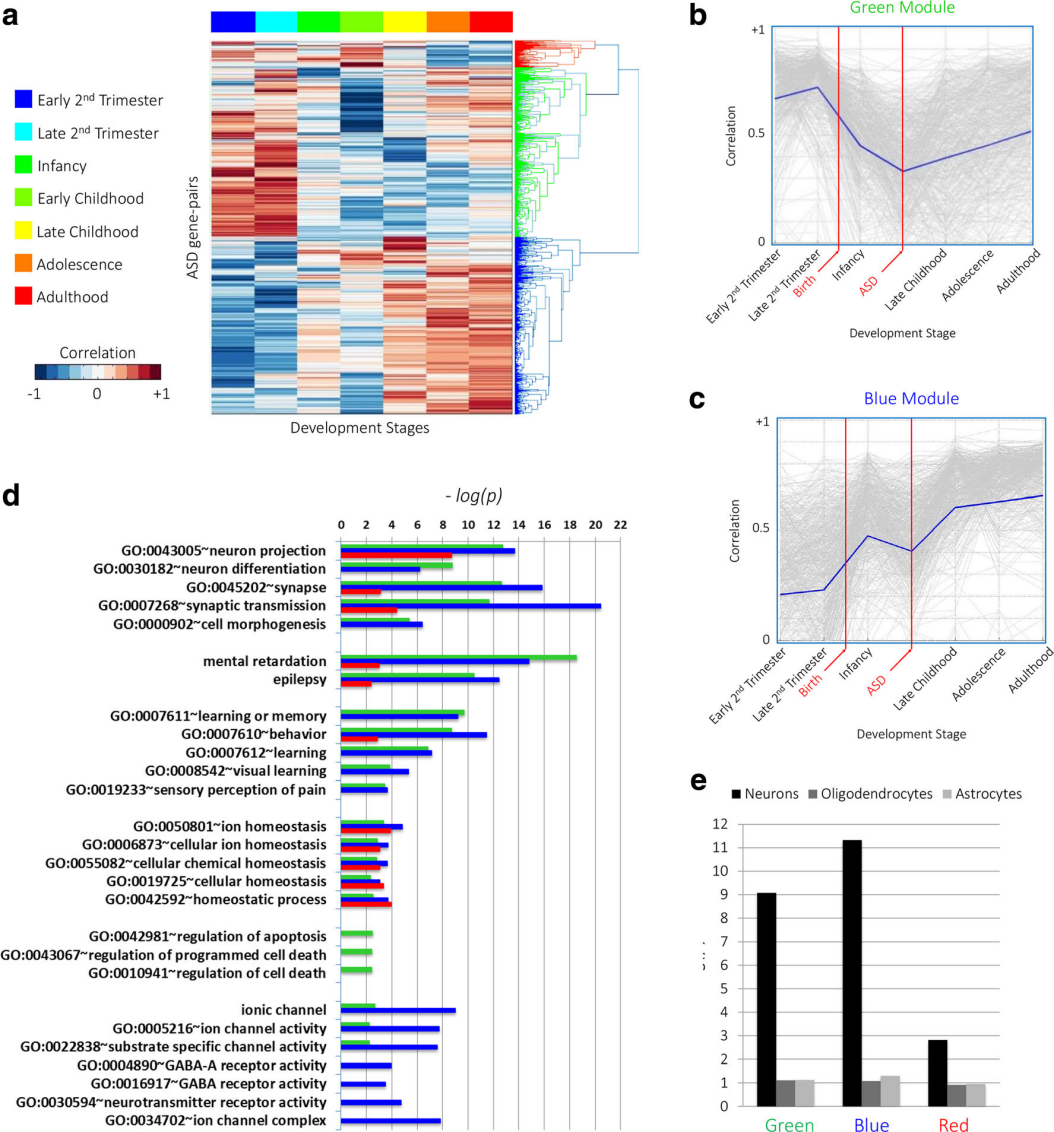
Spatio-temporal gene co-expression analysis of ASD candidate genes. **a** Heatmap of the temporal correlation pattern of ASD gene pairs (*rows*) through different developmental stages (*columns*). The dendrogram to the right shows the clustering of ASD gene pairs into three modules (*red*, *green*, and *blue*). **b** The average correlation pattern of gene pairs in the green module shows loss of correlation at childhood. *Vertical lines* indicate birth and average age of ASD diagnosis. **c** The average correlation pattern of gene pairs in the blue module shows progressive gain of correlation across development. **d** Gene ontology terms enriched in each of the three modules (represented in −*log*_10_(*p*), *Benjamini-corrected*). *Bars* are colored according to the module’s name. **e** Enrichment scores for each of the ASD modules in neurons, astrocytes, and oligodendrocytes (represented in −*log*_10_(*p*), *FDR-corrected*)

Figure 2a shows the hierarchical clustering of the retained ASD gene pairs. In total, there were 103,285 pair-wise correlations between the 455 ASD candidate genes in the ASD list, of which 1168 remained after applying the stringent threshold of an absolute correlation *greater than 0.8*. The surviving gene pairs clustered into three distinct modules. Two of these modules, the “green” module and the “blue” module, displayed distinct correlation patterns relative to pre- versus postnatal development. The green module (Fig. 2b) consisted of gene pairs that lose correlation in the middle stages of development (infancy and childhood); that is, each pair of genes within the green module has highly correlated spatial expression profiles at prenatal developmental stages, but this correlation is lost at birth. In contrast, the blue module (Fig. 2c) consisted of gene pairs that gain correlation during development. These genes do not show correlation at prenatal stages but progressively increase correlation throughout postnatal development. The “red” module did not show any coordinated pattern of expression over developmental time (Fig. S1). Genes forming gene pairs in each of the three modules are listed in Table S1.

To characterize these modules further, we used the gene ontology (GO) enrichment analysis tool DAVID 6.7 (Huang et al. 2009) to discover whether genes in these modules relate to specific molecular mechanisms, cellular pathways or disease annotation terms. The top significantly enriched terms (Benjamini-corrected *p* values <0.01) are summarized as shown in Fig. 2d. All the three modules were enriched for annotation terms related to *neuron projection*, *synapse*, *synaptic transmission*, and *behavior*. The three modules were also enriched for disease terms including *mental retardation* and *epilepsy*. The green and blue modules were significantly enriched for *neuron differentiation*, *cell morphogenesis*, and *learning*/*memory*. The green module was specifically enriched in functional terms related to *regulation of apoptosis* and *regulation of cell death*, while the blue module was specifically enriched in terms related to *ion channel*, *neurotransmitter receptor activity*, and *GABA receptor activity*. Table S2 includes the full list of enriched gene-annotation terms for these two modules.

None of the GO terms that were significantly enriched in the three ASD modules showed any significant enrichment in modules from ten randomly created sets (see Table S3). We also assessed how many gene pairs remained after thresholding them on co-expression (absolute correlation >0.8 at any developmental stage) in 10,000 random gene sets of 455 genes. The results are summarized in Fig. S2, where we show that the number of gene pairs remaining after thresholding the ASD list (1168 gene pairs) is highly significant (*p* < 10^−4^).

#### Modules of ASD Candidate Genes Are Enriched in Neurons

We then assessed if these modules were enriched in specific brain cell types. Lists of cell-type-specific genes were obtained from a previously published work (Cahoy et al. 2008). These lists included 1465 neuron-, 1529 oligodendrocyte-, and 1829 astrocyte-specific genes (Table S4). ASD candidate gene modules were assessed for enrichment of these cell types using the hypergeometric probability test (see “Material and Methods”). Both the green and blue modules were significantly enriched in neurons, whereas the red module demonstrated no significant enrichment, as shown in Fig. 2e.

### Enrichment of ASD Candidate Genes in Transcriptome-Wide Molecular Interaction Modules

Given the marked genetic heterogeneity of ASD and the large number of genes involved, it is also important to understand the role of ASD candidate genes in normal brain development within the context of the whole transcriptome, as subnetworks of the entire brain transcriptome may be perturbed by the ASD candidates. An analysis of these subnetworks could reveal ASD-related pathways that would be missed by analyzing the ASD candidates alone, as it is unlikely that all ASD candidate genes have been identified to date (Sanders et al. 2012). Moreover, this top-down approach allows the identification of other genes that might also relate to ASD. Therefore, we performed a transcriptome-wide co-expression network analysis to identify functionally related gene modules throughout the normal developing brain transcriptome (“molecular interaction modules”). Then, we assessed whether these modules were specific to distinct brain regions or developmental stages, and if they were related to specific pathways, cellular processes, or disease annotation terms. Finally, we determined if ASD candidate genes were enriched in any of the resultant molecular interaction modules.

#### No Evidence for Region-Specific Modules

**Fig. 3:**
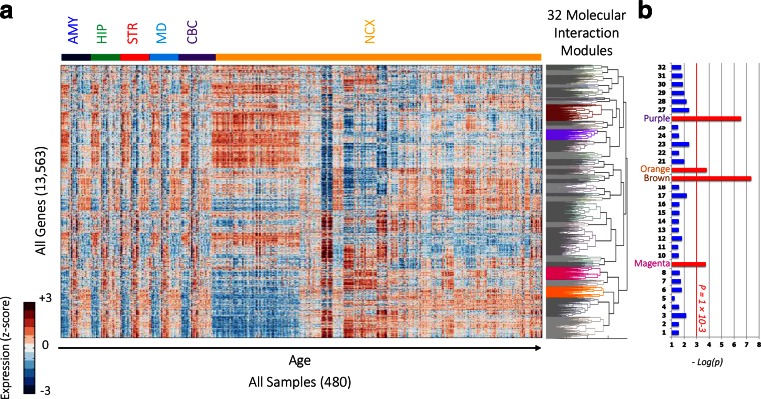
Transcriptome-wide molecular interaction network. **a** A heatmap of the expression of 13,563 genes (*rows*) across all 480 samples (*columns*). Samples are ordered first by brain region (*color-code at the top*) and then by age. The dendrogram to the *right* shows the clustering of all the genes into 32 modules. Modules with significant enrichment (*p* < 10^−*3*^) of genes from the ASD list are *colored* while other modules are shown in *gray*. **b** Enrichment of ASD candidate genes in each of the modules showing high significance in the *magenta*, *brown*, *orange*, and *purple* modules (represented by −*log*_10_(*p*), *FDR-corrected*)

The transcriptome-wide co-expression network was constructed from all genes expressed in the brain (13,563 genes), based on their expression profile across all samples (480 samples, i.e., all brain structures and developmental stages). Genes were hierarchically clustered based on Spearman’s rank correlation and complete linkage between pairs of genes. The resulting network consisted of 32 modules of varying size (from 36 to 1386 genes), as shown in Fig. 3a. Visual analysis of the heatmap and average expression patterns of member genes from each of the 32 modules demonstrated that none were specific to particular anatomical regions. This observation is consistent with the results from a similar dataset of human brain development assessed by microarray (Ben-David and Shifman 2012a). We did not observe any pre/postnatal-specific expression patterns in any of the 32 modules (Fig. S3). The genes comprising each of the 32 modules are listed in Table S5.

#### Modules Enriched for ASD Genes Relate to Synaptogenesis, Protein Turnover, and Mitochondria

**Fig. 4:**
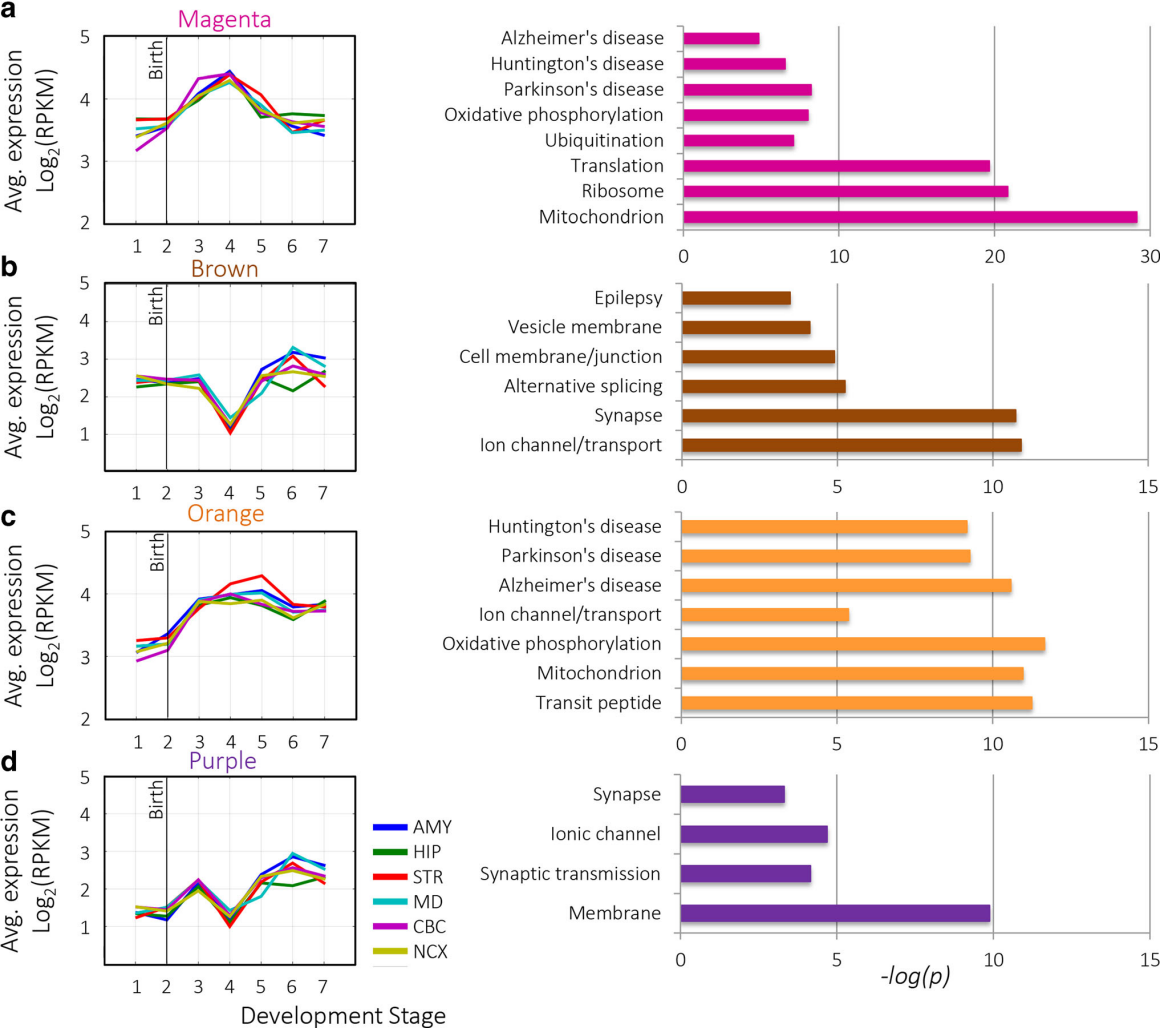
ASD modules. **a** *Left*, average expression pattern of the magenta module genes across different brain regions (*different plot colors*). *Right*, top GO terms enriched in the magenta module. **b** *Left*, average expression pattern of the *brown* module genes. *Right*, top GO terms enriched in the *brown* module. **c** *Left*, average expression pattern of the *orange* module genes. *Right*, top GO terms enriched in the *orange* module. **d** *Left*, average expression pattern of the *purple* module genes. *Right*, top GO terms enriched in the *purple* module. All enrichment values are represtented by −*log*_10_(*p*), *Benjamini-corrected*

The resulting transcriptome-wide co-expression modules were then assessed for enrichment of genes belonging to the ASD list using the hypergeometric probability test. Four modules—magenta, brown, orange, and purple—were significantly enriched for ASD candidate genes (FDR-corrected *p* values <0.001), as shown in Fig. 3b. The magenta module (Fig. 4a) contained highly co-expressed genes during early childhood. The brown module (Fig. 4b) included genes with weak co-expression during childhood and differential spatial co-expression at late developmental stages. The orange module (Fig. 4c) contained genes with progressively increasing co-expression during development. Finally, the purple module (Fig. 4d) included genes with varied co-expression during development and high differential spatial co-expression in adolescence and adulthood.

Then, these ASD-enriched modules were tested for enrichment of gene ontology terms, as shown in Fig. 4 (see Table S6 for full list). The magenta and orange modules were significantly enriched for *mitochondrial* processes. Additional GO terms that were significantly enriched in the modules included *ribosome* and *protein translatio*n, *transit peptide*, *ubiquitination*, and *alternative splicing*. Significant enrichment for *synapse* was also found in the brown module and the purple module. Enrichment of ASD candidate genes into transcriptome-wide synapse modules further supports our previous finding of ASD modules (green and blue modules), above, which were also related to synaptogenesis. Neurological disease terms were also significant in the ASD-enriched modules: *epilepsy* (brown module), *Parkinson*’*s* (magenta and orange modules), *Alzheimer*’*s* (magenta and orange modules) and *Huntington*’*s* (magenta and orange modules).

#### ASD-Enriched Molecular Interaction Modules Are Mainly Neuronal

**Fig. 5:**
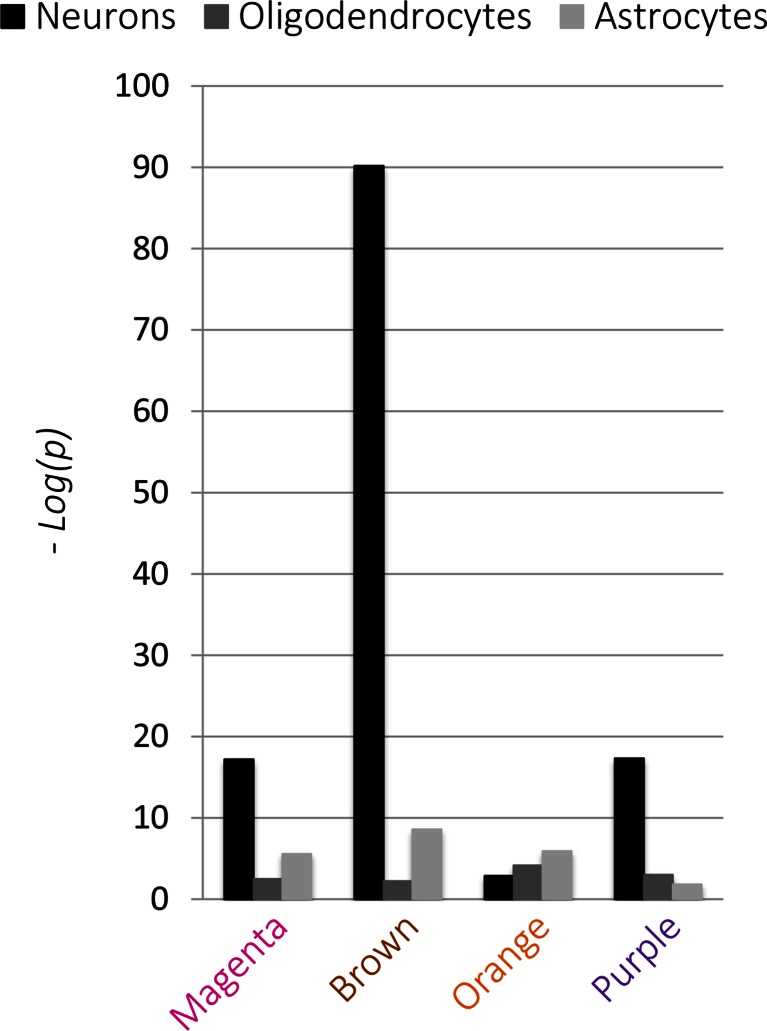
Enrichment of the ASD modules in cell-type-specific genes. Enrichment of ASD-enriched modules in neurons, oligodendrocytes, and astrocytes (represented in −*log*_10_(*p*), *FDR-corrected*)

Each module was also tested for enrichment of specific neural cell populations (i.e., neurons, oligodendrocytes, and astrocytes), as described earlier. Three out of the four ASD-enriched modules were enriched for neurons (*magenta*, *brown, and purple modules*), as shown in Fig. 5. The orange module, which was related to mitochondrial functioning, was highly enriched in astrocytes but not neurons. This finding is of relevance, as multiple recent studies have implicated glia, and specifically astrocytes, in the brain pathology of autistic subjects (Lioy et al. 2011; Cao et al. 2012).

#### ASD-Enriched Molecular Interaction Module Hub Genes Provide Molecular Targets

**Fig. 6:**
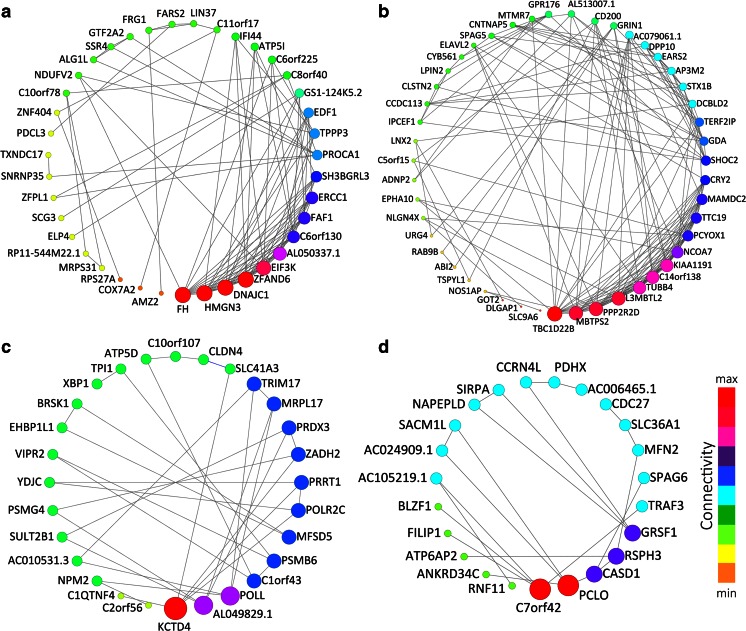
Hub genes of ASD modules. Each of the four ASD-enriched modules is presented with the degree-sorted circle layout of cytoscape, with the nodes’ size and color reflect the level of connectivity within the network. The bigger the node, the more connections it has. For clarity, edges with correlation values smaller than 0.9 are removed. **a** Top-connected genes of the *magenta* module. **b** Top-connected genes of the *brown* module. **c** Top-connected genes of the *orange* module. **d** Top-connected genes of the *purple* module

An alternative approach to annotate the function of each ASD-enriched module is to analyze the genes with the strongest correlations within each module. It has been shown that within an interaction network, genes with the most connections to other genes, termed hub genes, are informative for the network as a whole, and are potential high-yield therapeutic targets (Barabási et al. 2011). The strongest correlations within a module were explored using Cytoscape v2.8 (Smoot et al. 2011). First, each ASD-enriched module (magenta, brown, orange, and purple) was imported as a graph with genes acting as nodes and pair-wise correlations between genes representing edges between the nodes. Figure 6 shows a subset of the connected nodes within each graph.

The ten most highly connected nodes (genes) within each graph were extracted and their putative functions determined by manual curation of the literature. Among these most highly connected hub genes, a number were of note. The most striking observation was that most of the highly connected hub genes in the magenta and brown modules are known to function in the processes of *chromatin remodeling*, *transcription*, or *translation* (*HMGN3*, *EIF3K*, *ZFAND6*, *DNAJC1*, *C6orf130*, *ERCC1*, *LCMBT2*, *MBTPS2*, *KIAA1191*, *C14orf138*, *GDA*, and *NCOA7*). This result is in line with the gene ontology enrichment for these modules (Fig. 4). A number of other central hub genes are involved in *intracellular signaling pathways* (*PROCA1*, *TBC1D22B*, *PPP2R2D*, *HACE1*) and a few are known to function as *membrane ion channels* (*PRRT1*, *KCTD4*, *SLC26A1*, *KCNA4*). In addition, a number of hub genes function in *apoptosis* or *myeloid*/*microglia* cell processes (such as *RNF11*, *CD200*, and *FAF1*). These hub gene functions largely recapitulate the ontologies of their respective networks, supporting our enrichment results and highlighting potential critical regulatory molecules of these networks.

## Discussion

In order to gain insight into the molecular pathogenesis of ASD, we present a biologically driven computational approach to analyze a heterogeneous set of genes previously independently implicated in ASD, to understand if they may relate to each other through shared functional genomics mechanisms. The main goal of this work is to understand if ASD candidate genes relate to common cellular/molecular pathways when considered in the context of transcription during normal human brain development. Identifying such pathways has profound implications for understanding the pathophysiology of ASD, especially since the majority of ASD patients do not have an identifiable genetic mutation (Huguet et al. 2013). Yet, those patients are still likely to have alterations in the same pathways that are affected as those ASD patients with genetic mutations, although the alterations may be caused by environmental, epigenetic, or other non-genetic factors.

We intentionally analyzed a very broad collection of genes associated with ASD, in an attempt to understand if there are cellular or molecular pathways that may represent final common mechanisms across all patients. Despite the fact that some of the genes in our ASD list are essentially causative for ASD (for instance, single gene mutation syndromes such as Fragile X), while others are not as strongly associated, we have weighted all genes equally to avoid bias toward more severely affected patient cases. Future work could attempt to weigh genes differently within the co-expression networks to study different genetic subtypes of autism.

We discovered subsets of ASD candidate gene modules that displayed biologically relevant co-expression dynamics, which were enriched for the processes of synaptogenesis, apoptosis, and GABA-ergic signaling. In addition, we assessed for functional genomic relationships between ASD candidate genes and the entire developing human brain transcriptome. This analysis revealed that ASD candidate genes are enriched within transcriptome-wide modules related to synaptogenesis, mitochondrial function, alternative splicing, protein translation, and ubiquitination. By identifying gene modules that have similar expression patterns in the brain (regardless of time period), we were able to infer that they are likely functioning in similar pathways. This allowed us to infer which cellular and molecular mechanisms are likely to be disrupted in autism. We also demonstrated the cell-type-specific enrichment of these modules being mostly neurons, further supporting the biological relevance of our computational approach, as the broad ASD phenotype is generally consider to ultimately result from neuronal/synaptic abnormalities (Zoghbi 2003). Although several brain regions have been highlighted in neuroimaging and connectivity studies of autistic brains (namely cortical regions and the cerebellum) (Carper and Courchesne 2005; Courchesne and Pierce 2005), interestingly, none of the transcriptome-wide modules were specific to particular anatomical regions, which supports previous reports of the BrainSpan dataset via microarray (Kang et al. 2011). Finally, by assessing genes with the highest connectivity within the transcriptome-wide molecular interaction modules that were enriched for ASD candidates, we identified hub genes that may represent critical regulatory molecules in these networks, and their functions further supported our enrichment findings.

The number of strongly connected gene pairs from the ASD list were found to be highly significant (*p* = 10^−4^), indicating that—based on their significantly strong co-expression across development—those ASD-associated genes are likely to be functionally related. We discovered three subsets of ASD-associated genes with distinct co-expression profiles around birth, even though the co-expression network for each developmental stage was calculated separately to avoid any bias toward pre/postnatal expression changes. All three of these modules were significantly enriched for the processes of synaptogenesis and behavior, in addition to the disease annotations of mental retardation and epilepsy. Two of the modules (the green and blue modules) were also significantly associated with cell morphogenesis, neuron differentiation, and learning. Moreover, the green module, which had highly correlated spatial expression at prenatal developmental stages with a dramatic loss of correlation at birth, was uniquely enriched for the process of apoptosis. Conversely, the blue module displayed an opposite co-expression trajectory—poor correlation in expression prior to birth, but strong co-expression beginning in infancy and increasing through adulthood—and was uniquely related to GABA-ergic signaling and ion channels. The distinct, biologically relevant expression patterns of these two modules around birth, a developmental period with the greatest shifts in gene expression (Kang et al. 2011), suggests a key role of these networks in brain development and autism.

ASD-associated genes were highly co-expressed later in development in some of the identified modules (childhood and adulthood), whereas autism symptoms are generally apparent by the age of two. Our results suggest that a heterogenous set of genes which were independently associated to ASD converge into few functional pathways late in normal development. However, our findings do not preclude the possibility that the pathways implicated by these modules are involved in ASD pathogenesis, as our analysis was on co-expression patterns, not absolute gene expression levels. It is possible that the genes in these modules are still expressed in early neurodevelopment but that they are most strongly co-expressed with other genes in the same module later in life. Consequently, disruption of the integrity of these genes (through inherited mutations, de novo mutations, mis-expression, etc.) early in development is likely to disrupt the functions of those modules later in life.

The functional ontologies of these networks are all pathways previously implicated ASD. Disrupted synaptogenesis has been one of the most replicated findings in ASD research (Bourgeron 2009), and autism is largely considered to be a disorder that results from a convergence of factors into synaptic dysfunction (Zhoghbi 2003). Our finding of multiple ASD gene co-expression networks enriched for the function of synaptogenesis is in line with these previous studies. Additionally, our analysis shows these same transcriptional networks are related to the processes of GABA-ergic signaling and apoptosis, which have been independently associated with ASD through various approaches. GABA-ergic neurons are the main inhibitory cells of the brain, and much research has suggested that an imbalance in the ratio of inhibitory to excitatory neurons may underlie autism at the cellular circuit level (Rubenstein and Merzenich 2003). Furthermore, a number of clinical trials are currently ongoing to test GABA-ergic modulators for the treatment of ASD (Spooren et al. 2012). Likewise, apoptosis—and more specifically the pruning of overabundant neural connections in early development—has recently been shown to be a critical process in the developing mammalian brain (Paolicelli et al. 2011), and a number of studies have suggested this process may be aberrant in ASD (Sheikh et al. 2010; Maezawa et al. 2011). A delicate balance between formation of needed synaptic connections and pruning of overabundant connectivity (and their excitatory/inhibitor ratio) is a main component of early experience-dependent brain development, and both human and animal studies have previously shown deficiencies in these processes in ASD (Courchesne and Pierce 2005). Our results suggest these processes may relate to each other and to ASD candidate genes through shared transcriptional networks.

ASD candidate gene modules with distinct temporal co-expression profiles around birth, which are highly related to synaptogenesis, support the notion that the pathogenesis of ASD is strongly related to this process. Additionally, the demonstration that the same transcriptional networks are also related to GABA-ergic signaling and apoptosis—both also suggested to be aberrant in autism—suggests that these disparate pathways may relate to each other through underlying shared transcriptional networks, providing a potential mechanism for functional convergence of ASD candidate genes into common pathways underlying autism.

By incorporating the ASD candidate genes into the context of the entire brain transcriptome, our results suggest that the disruption of synaptogenesis in autism may also relate to underlying basic cellular processes—alternative splicing, protein translation, and ubiquitination—which have previously been implicated in ASD (Kelleher and Bear 2008; Glessner et al. 2009; Smith and Sadee 2011; Piton et al. 2012). Defects in protein translation in particular have recently been shown to be a prominent feature in multiple animal models of ASD (Neves-Pereira et al. 2009; Gkogkas et al. 2013; Santini et al. 2013).

Two transcriptome-wide modules that were enriched for ASD candidate genes were both related to mitochondrial function, and one was specifically enriched in glia but not neurons. A large body of evidence has associated mitochondria dysfunction with rare syndromic forms of autism (Rossignol and Frye 2012) and recent evidence suggests that altered mitochondrial gene expression may contribute to non-syndromic autism as well (Anitha et al. 2012a, b). Furthermore, these modules were also related to Huntington’s and Alzheimer’s disease, both known to have mitochondrial defects associated with their pathogenesis (Sheng and Cai 2012). While the ASD-only gene modules in the first part of this study did not implicate mitochondrial function, significant enrichment of ASD genes in two different transcriptome-wide networks related to mitochondria suggests that additional ASD genes related to mitochondria may remain to be discovered, and our hub gene analysis provides potential high confidence candidates.

While the phenotype of autism may ultimately result from dysfunctional synaptogenesis, it is possible that such fundamental cellular processes as protein translation, ubiquitination, alternative splicing, and mitochondrial function may underlie the synaptic dysfunction. Furthermore, this may help explain the incredibly variable clinical spectrum of autism and account for the increased prevalence of other complex medical problems in both the brain and other systems that ASD patients experience (Levy et al. 2009). Moreover, a recent meta-analysis of de novo mutations in autism demonstrated enrichment for genes related to transcriptional regulation and showed they have similar neurodevelopmental expression patterns to the green and blue modules of ASD candidates we identified (Ben-David and Shifman 2012a). Multiple recent whole-exome sequencing studies of individuals and family trios have continued to support the role of transcription- and synaptogenesis-related genes in ASD (Bernier et al. 2014; De Rubeis et al. 2014; Iossifov et al. 2014). Furthermore, a similar network analysis approach that assessed specifically for enrichment of de novo variants implicated in ASD and intellectual disability found similar shared transcriptional networks (Hormozdiari et al. 2015). By integrating co-expression and protein-protein interaction networks they demonstrated that ASD-related genes converge into two modules related to basic intracellular processes including transcriptional regulation and synaptogenesis, and that the former process was more operant in prenatal time periods and the later in postnatal development (Hormozdiari et al. 2015). These results are in line with earlier findings using either co-expression networks only (Parikshak et al. 2013, Willsey et al. 2013) or protein-protein interaction networks only (Pinto et al. 2014). Our results, despite assessing a much broader set of ASD candidate genes, are largely in agreement with these recent results. Whether and how defects in these basic cellular mechanisms result in altered synaptogenesis, are a reaction to altered synaptogenesis, or are mutually exclusive from synaptogenesis is unclear. However, our results in addition to these previous studies suggest that a complex interplay between these processes and synaptogenesis are related to each other through overlapping co-expression networks.

A number of studies have assessed for changes in gene expression in postmortem autistic brain directly (for a review, see, Lintas et al. 2012; Voineagu 2012). These studies have repeatedly shown that the autistic transcriptome is abnormally expressed compared with control brains across many different brain regions. The genes that are mis-expressed in autistic brains have been consistently demonstrated to be involved in pathways related to the synapse (Voineagu et al. 2011; Chow et al. 2012), immune response/apoptosis (Garbett et al. 2008; Voineagu et al. 2011; Chow et al. 2012), neurotransmitter receptors (Purcell et al. 2001), RNA splicing (Voineagu et al. 2011; Ziats and Rennert 2013; Chow et al. 2012), and mitochondrial function (Smith et al. 2012; Anitha et al. 2012b). These findings in autistic brain complement our results by showing that the ASD co-expression modules we discovered in the *normal* developing brain are functioning in the same pathways that are consistently disrupted in autistic brains.

Finally, the identified hub genes of ASD-enriched modules recapitulate the gene ontology analysis of these modules, strengthening the observation that basic cellular functions related to genome processing and mitochondrial function may represent a nexus in the genomic pathology of ASD. In addition, a number of hub genes relate to myeloid cells and apoptosis. There is a growing body of evidence implicating cytokine signaling, microglia-mediated synaptic pruning, and other immune-related processes in ASD (Maezawa et al. 2011), and this finding suggests the autism candidate genes may indirectly relate to processes that interact with these pathways through the transcriptional machinery. Furthermore, this supports our finding that the green module of autism candidate genes relates to apoptotic pathways. However, because comprehensive lists of microglia-specific marker genes are not available, we were unable to assess for enrichment of ASD candidate genes into this cell type in this study. By highlighting individual genes that are most central in the identified molecular interaction networks, the hub gene analysis may provide potential additional high-yield ASD candidates for their respective transcriptional networks.

In summary, we have profiled the transcriptional co-expression networks of autism candidate genes throughout normal human brain development to identify modules of ASD candidate genes with biologically relevant expression patterns. We have shown that these ASD modules are enriched for synaptogenesis, apoptosis, and GABA-ergic signaling, suggesting that pathways previously independently implicated in autism are related to each other through shared neurodevelopmental transcriptional networks. In addition, we expanded the analysis of ASD candidates to consider their relationship with the entire brain transcriptome. We demonstrated that ASD-enriched transcriptome-wide molecular interaction modules are related to mitochondrial function, splicing, and protein turnover, which suggests further ASD candidates related to these functions may remain to be discovered.

Our comprehensive analysis of the global co-expression relationships between ASD candidates demonstrates that the various pathways implicated in autism separately may relate to one another when considered in a broader functional genomics framework. Furthermore, our molecular interaction module analysis represents a valuable strategy to identify and prioritize other potential ASD candidate genes. Moreover, this approach can be used to assess genes from other complex neurodevelopmental and psychiatric disorders like schizophrenia, to uncover potential overlapping transcriptional pathways in the developing human brain among other gene sets.

## Material and Methods

### Dataset Summary

#### Developing Human Brain Transcriptome Data

We downloaded the *BrainSpan* transcriptional atlas from http://www.brainspan.org. Details of tissue acquisition and data processing can be found in the *BrainSpan* documentation. The atlas contains next-generation RNA sequencing (RNA-seq) data generated from 579 tissue samples. These samples were collected from 41 developing and adult postmortem brains of neurologically unremarkable donors spanning early pre-natal development (8 postconception weeks (PCW)) to late adulthood (40 years of age).

Some donor brains in the *BrainSpan Atlas* have missing data from certain brain regions. We excluded donors that had more than six regions missing. For donors with six or less missing regions, we imputed the data for the missing brain regions using a nearest neighbor approach. A full mathematical description of this is provided in the Supplementary Information. The resulting dataset contained 30 donor brains. From these donor brains, only the 16 brain regions that were present in all 30 donor datasets were analyzed. This filtration resulted in a final dataset derived from 30 donor brains across 16 brain regions, or 480 brain samples in total.

The 30 donor brains used in our analysis were further grouped into seven developmental stages according to the *BrainSpan* classification system (Fig. 1a). The transcriptomes of the cerebellar cortex (CBC), medial-dorsal nucleus of the thalamus (MD), striatum (STR), amygdala (AMY), hippocampus (HIP), and 11 areas of the neocortex (NCX) were assessed (Fig. 1b).

The resultant dataset contained RNA-seq expression values aligned to composite gene models, and given in units of reads per kilobase of exon model per million mapped reads (RPKM) (Mortazavi et al. 2008). Genes whose RPKM values were likely to represent noise rather than actual sequenced reads were discarded by removing any gene that did not have at least one expression value greater than or equal to five RPKM in any of the 480 tissue samples. The remaining set consisted of 13,563 genes expressed in the 30 donor samples assessed. The expression data was then normalized across all samples using quintile normalization. Finally, the data was log_2_-transformed for further analysis.

#### ASD Gene List

A comprehensive yet high confidence list of common ASD susceptibility genes (herein named “ASD list”) was created by combining (taking the union) lists from three main ASD genes databases: AutDB (Basu et al. 2009), Autism Genetics Database (AGD) (Matuszek and Talebizadeh 2009), and AutKB-484 (Xu et al. 2012) (a subset of AutKB determined by Xu et al. through ranking and scoring algorithm to be the most high-confidence ASD candidates). These databases each independently collected genes that have previously been associated with autism through a number of different experimental studies using various methods (namely GWAS, single-gene deletion syndromes that have autism as a component, genome-wide expression profiling, and genome-wide sequencing/CNV/linkage studies). ASD genes that were not present in the 13,563 genes we considered from the *BrainSpan* atlas (for instance, mitochondrially encoded genes) were discarded. The final ASD list consisted of 455 ASD susceptibility genes (Table S7).

#### Co-expression of ASD Candidate Genes

We calculated the Spearman’s rank correlation between each pair of ASD candidate genes within each of the seven developmental stages separately. For each donor, the correlation between each gene pair is calculated across all 16 brain regions. The correlation between a gene pair in each developmental stage is the average of their correlation across all donors within the developmental stage. We focused our analysis on gene pairs with an absolute correlation value greater than 0.8 in at least one developmental stage (1168 out of 103,285 gene pairs). We used hierarchical clustering to cluster gene pairs using the Euclidean distance between the profiles and a complete linkage to merge clusters. Based on the heatmap of gene pair correlations across development, we cut the dendrogram to produce three clusters. The correlation pattern for each module was summarized by averaging all the gene pair correlation patterns included in the respective module.

#### Transcriptome-Wide Co-expression Network

We constructed a transcriptome-wide co-expression from all genes expressed in the brain (13,563 genes), based on the similarity of their expression profile across all samples (480 samples). We used hierarchical clustering to cluster gene pairs using Spearman’s rank correlation between the profiles and a complete linkage to merge clusters. We cut the dendrogam to produce 32 modules of varying size (from 36 to 1386 genes).

#### Gene Set Enrichment and Gene Ontology Enrichment Analysis

Enrichment of transcriptome-wide molecular interaction modules for ASD candidate genes and cell-type-specific genes was assessed using the hypergeometric probability density function (hygepdf) in MATLAB R2011a (The MathWorks, Inc.). The resulting *p* values were corrected for multiple testing using false discovery rate (FDR). All results reported are the −*log*10 of FDR-corrected *p* values, and only *p* values <0.001 were considered significant.

Gene list were assessed for shared biological pathways by testing for enrichment of gene ontology terms (GO) using DAVID Bioinformatics Resources 6.7 (Huang et al. 2009). The complete list of expressed genes in this study’s dataset (13,563 genes) was used as the background. Only gene ontology terms with a Benjamini-Hochberg multiple testing-corrected *p* value <0.01 are presented as significant.

## Electronic supplementary material

Fig. S1 *Red module*. The average correlation pattern of gene pairs in the red module does not show any coordinated pattern of expression across development. *Red lines* indicate birth and age on ASD diagnosis. (PDF 69 kb)

Fig. S2 Distribution plot of the number of strongly correlated gene pairs per gene set. The distribution of the number of gene pairs remaining after applying the threshold (absolute correlation >0.8 at any developmental stage) shows that that the number of strongly correlated gene pairs from the ASD list (dashed red line) is highly significant (*p* = 10^−4^). *Blue bars* correspond to the 10,000 random gene sets analyzed. (PDF 92 kb)

Fig. S3 Average expression patterns of the 32 molecular interaction networks. Each graph shows the average expression pattern of all the genes within one of the 32 modules resulting from the transcriptome-wide co-expression network. *Different colors* represent different brain structures. (PDF 270 kb)

Supplementary Information Further information regarding the details of our methods including: handling missing data, Spearman’s rank correlation, and random control networks. (PDF 159 kb)

Table S1 Modules of ASD candidate genes. A table listing member genes of the *green*, *blue*, and *red* modules. (XLS 60 kb)

Table S2 GO enrichment analysis of ASD candidate genes. A table listing all the significantly enriched (Benjamini-adjusted *p* value <0.01) GO terms in each of the ASD co-expression modules. (XLS 202 kb)

Table S3 GO enrichment analysis of ten random gene sets. A table listing the GO enrichment analysis of the co-expression modules created by clustering ten random sets of 455 genes. Significantly enriched terms (Benjamini-adjusted *p* value <0.01) are highlighted in *green*. (XLSX 402 kb)

Table S4 Cell-type-specific genes. A table listing genes enriched in specific cell-types (i.e., neurons, oligodendrocytes and astrocytes) as extracted from (Cahoy et al. 2008). (XLS 400 kb)

Table S5 Modules of whole-genome molecular interaction network. A table listing member genes of the 32 modules of the transcriptome-wide molecular interaction network. (XLS 990 kb)

Table S6 GO enrichment analysis of transcriptome-wide molecular interaction modules. A table listing all the significantly enriched (Benjamini-adjusted *p* value <0.01) GO terms in each of the transcriptome-wide molecular interaction modules. (XLS 1153 kb)

Table S7 ASD list. A table listing the 455 genes forming the list of ASD candidate genes in our study. (XLS 64 kb)
