# Supplementary material for: Shared Pathways Among Autism Candidate Genes Determined by Co-expression Network Analysis of the Developing Human Brain Transcriptome
Source: J Mol Neurosci. 2015 Sep 23;57(4):580–94. doi: 10.1007/s12031-015-0641-3 (PMC4644211; doi:10.1007/s12031-015-0641-3)
Supplement: Supplementary file 1 — Red module. The average correlation pattern of gene pairs in the red module does not show any coordinated pattern of expression across development. Red lines indicate birth and age on ASD diagnosis. (PDF 69 kb) [file 12031_2015_641_MOESM1_ESM.pdf]

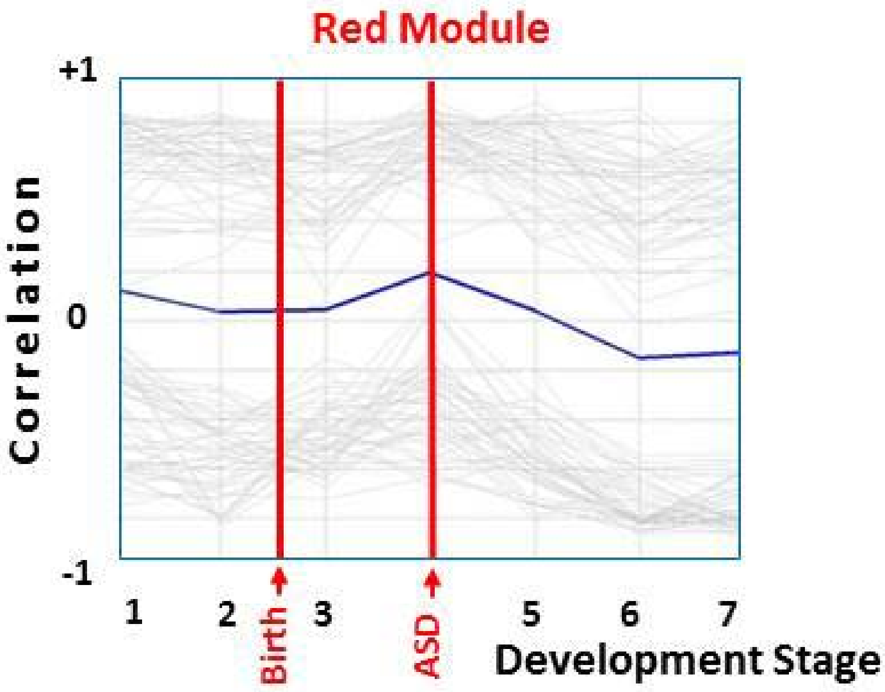

Figure S1 | Red Module. The average correlation pattern of gene-pairs in the red module does not show any coordinated pattern of expression across development. Red lines indicate birth and age on ASD diagnosis.
