# Supplementary material for: Shared Pathways Among Autism Candidate Genes Determined by Co-expression Network Analysis of the Developing Human Brain Transcriptome
Source: J Mol Neurosci. 2015 Sep 23;57(4):580–94. doi: 10.1007/s12031-015-0641-3 (PMC4644211; doi:10.1007/s12031-015-0641-3)
Supplement: Supplementary file 2 — Distribution plot of the number of strongly correlated gene pairs per gene set. The distribution of the number of gene pairs remaining after applying the threshold (absolute correlation >0.8 at any developmental stage) shows that that the number of strongly correlated gene pairs from the ASD list (dashed red line) is highly significant (p = 10−4). Blue bars correspond to the 10,000 random gene sets analyzed. (PDF 92 kb) [file 12031_2015_641_MOESM2_ESM.pdf]

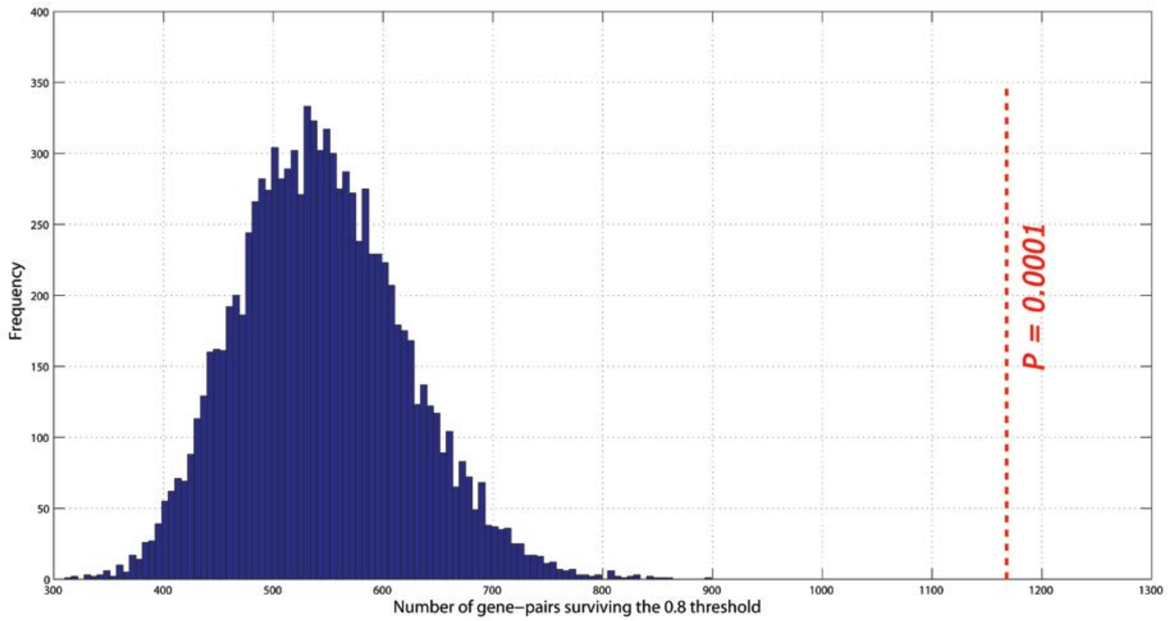

Figure S2 | Distribution plot of the number of strongly correlated gene-pairs per gene set. The distribution of the number of gene-pairs remaining after applying the threshold (absolute correlation > 0.8 at any developmental stage) shows that that the number of strongly correlated gene-pairs from the ASD list (dashed red line) is highly significant ($p = 10^{-4}$). Blue bars correspond to the 10,000 random gene sets analyzed.
