# Supplementary material for: Shared Pathways Among Autism Candidate Genes Determined by Co-expression Network Analysis of the Developing Human Brain Transcriptome
Source: J Mol Neurosci. 2015 Sep 23;57(4):580–94. doi: 10.1007/s12031-015-0641-3 (PMC4644211; doi:10.1007/s12031-015-0641-3)
Supplement: Supplementary file 3 — Average expression patterns of the 32 molecular interaction networks. Each graph shows the average expression pattern of all the genes within one of the 32 modules resulting from the transcriptome-wide co-expression network. Different colors represent different brain structures. (PDF 270 kb) [file 12031_2015_641_MOESM3_ESM.pdf]

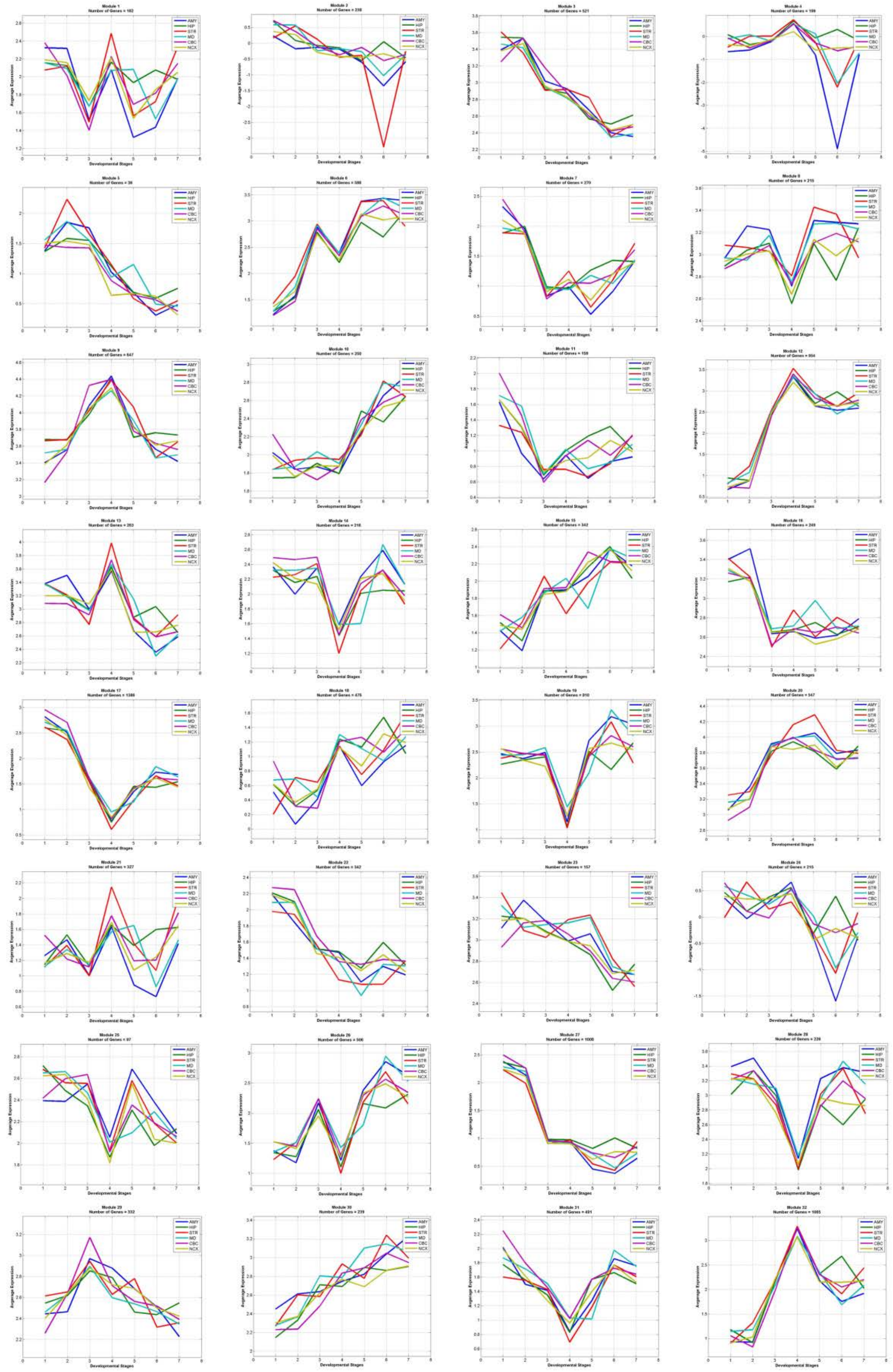

Figure S3 | Average Expression Patterns of the 32 Molecular Interaction Networks. Each graph shows the average expression pattern of all the genes within one of the 32 modules resulting from the transcriptome-wide co-expression network. Different colors represent different brain structures.
