# Supplementary material for: Shared Pathways Among Autism Candidate Genes Determined by Co-expression Network Analysis of the Developing Human Brain Transcriptome
Source: J Mol Neurosci. 2015 Sep 23;57(4):580–94. doi: 10.1007/s12031-015-0641-3 (PMC4644211; doi:10.1007/s12031-015-0641-3)
Supplement: Supplementary file 4 — Further information regarding the details of our methods including: handling missing data, Spearman’s rank correlation, and random control networks. (PDF 159 kb) [file 12031_2015_641_MOESM4_ESM.pdf]

Supplementary Information

In this Supplementary Information we provide further information regarding the details of our methods as well as some additional supplementary results.

Table of contents:

1. Supplementary Methods
    1.1. Handling Missing Data
    1.2. Spearman’s Rank Correlation
    1.3. Random Control Networks

1. Handling missing data

If donor ***A*** has a missing sample at structure ***S***, expression values for this sample are imputed in one of the following ways:

i. If there are other donors of the same age as donor *a*, namely b_i and $i = 1,\ldots k$ where *k* is the number of replicates (i.e. donors of the same age as donor *a*), then:

$$s_a = \frac{1}{k}\sum_{i\,\in\,k}\left(s_{b_i} + F_{b_i}^{a}\right)$$

where $F_{b_i}^{a}$ is defined as:

$$F_{b_i}^{a} = \frac{1}{r}\sum_{j\,\in\,r}\frac{1}{g}\sum_{l\,\in\,g}\log_2\left(\frac{Y_{jl}^{a}/N^{a}}{Y_{jl}^{b_i}/N^{b_i}}\right)$$

$F_{b_i}^{a}$ is a correction factor representing the average M-fold change between all samples of donor *a* compared to samples of donor b_i. *g* is the total number of genes; *r* is the number of brain structures. Y_{jl}^{a} and $Y_{jl}^{b_i}$ is the expression of gene *l* in structure *j* of donors *a* and b_i, respectively. N^{a} and N^{b_i} are the total sum of the expression values of donors *a* and b_i, respectively.

ii. If there is no other donor of the same age as donor *a*, we consider all the donors within the same age stage including donor *a* (e.g. Childhood). Donors of the age stage (excluding donor *a*) are: c_p and $p = 1,\ldots m$ where *m* is the number of donors in the age window of donor *a*, then:

$$s_a = \frac{1}{m}\sum_{p\,\in\,m}\left(s_{c_p} + F_{c_p}^{a}\right)$$

Where $F_{c_p}^{a}$ is the average M-fold change between all samples of donor *a* compared to the samples of donor c_j.

2. Spearman’s Rank Correlation

In our study, we used the Spearman’s Rank Correlation throughout to assess for pair-wise relationships among genes. Both the Spearman and Pearson correlation methods are measures of the strength of correlation between two variables. The difference is that Pearson's correlation assumes variables are normally distributed, whereas Spearman’s does not, and so is not affected by outliers or non-normal distributions. As a consequence, however, some precision in the data with regard to absolute values is lost when using Spearman.

We specifically chose the Spearman method for three reasons:

i. Having used a publicly available, large dataset, we were concerned about data normalization and noise. Although we applied a normalization step on the data before our analysis, the data was still produced from different brain donors, different brain regions, and each tissue sample was run in a separate sequencing experiment. This has the possibility of introducing technical variability in the absolute value of gene expression, which Pearson’s correlation would not correct for. As Spearman’s Rank Correlation focuses more on the similarity in the change of gene expression, as opposed to similarity in the absolute values of gene expression, we believed it would better account for possible technical variation in absolute gene expression levels.

ii. By considering the ranks rather than the actual expression values of gene-pairs (via Spearman’s correlation rather than Pearson’s), our results focus more on the similarity of expression value changes of each gene relative to all other genes, as opposed to similarities in the absolute expression values between genes. This is biologically relevant, as the biological consequence of a small change in a gene with a small dynamic range may be more important to the cell than a very large change in a gene with comparatively larger dynamic range (for a nice discussion of this issue, we would refer to Seita J, et al. Gene Expression Commons: an open platform for absolute gene expression profiling. PLoS One. 2012;7(7):e40321).

iii. By choosing to use Spearman’s Rank Correlation, our analysis was not focused on when gene pairs were highly expressed in absolute terms, but rather on when pairs of genes show similar patterns of change across developmental time. This allowed us to infer that genes may be regulated similarly because they are involved in shared transcriptional networks, and therefore when exactly they are expressed most highly is not a major focus of this study.

3. Random Control Networks

To assess the specificity of the results we obtained from the ASD list, we also generate two sets of random networks to compare our findings with. First, we generate 10 sets of 455 genes each, which were randomly selected from the 13,563 genes expressed in our BrainSpan dataset. These random gene sets were used to assess for the specificity of our gene ontology enrichment analysis of ASD candidates, as will be described later. None of the GO terms which were significantly enriched in the three modules showed any significant enrichment in modules resulting from 10 random sets analyzed, see Table S3.

Secondly, we created 10,000 random gene sets (again consisting of 455 genes each), and determined how many gene-pairs remained after applying the same thresholding we used to retain ASD gene-pairs, as will be described later. Results are shown in Figure S2.
